# Protective effects of chlorogenic acid on isoflurane‐induced cognitive impairment of aged mice

**DOI:** 10.1002/fsn3.2952

**Published:** 2022-06-20

**Authors:** Hao Chong, Yang Xi, Yan Zhou, Geng Wang

**Affiliations:** ^1^ Department of Anesthesiology Beijing Jishuitan Hospital Beijing China

**Keywords:** apoptosis, chlorogenic acid, cognitive impairments, inflammation, isoflurane, oxidative stress

## Abstract

Postoperative cognitive dysfunction (POCD) is characterized by impairment in cognitive functions in patients following anesthesia and surgery. Chlorogenic acid (CGA) is a plant‐derived compound possessing numerous bioactive properties. The aim of this study was to investigate the therapeutic potential of CGA in isoflurane (ISO)‐induced cognitive dysfunction of aged mice, and further identify the mechanisms involved in the protective effects of CGA. A total of 80 male C57BL/6 mice, 20‐month‐old, were randomly divided into control group, isoflurane group (ISO), and ISO + 30 mg/kg CGA group and ISO + 60 mg/kg CGA. CGA was given orally once daily for 7 days to the mice and they were exposed to ISO (1.5%; 4 h). The open‐field and Morris water maze tests were used to investigate the cognitive function of mice. Pretreatment with CGA significantly attenuated ISO‐induced cognitive impairment. The levels of IL‐1β, TNF‐α, IL‐6, nuclear p65 NF‐kB, cleaved caspase‐3, and Bax were significantly increased, while the levels of IkBα and Bcl‐2 were decreased in the hippocampus 24 h after the ISO anesthesia. All the mentioned effects induced by ISO were reversed by CGA pretreatment. Furthermore, ISO exposure induced marked downregulation of SOD, CAT, HO‐1, and NQO‐1 and elevation of MDA and nuclear translocation of Nrf2 in the hippocampus tissue. All these parameters were reversed by CGA treatment. Importantly, the higher dose of CGA (60 mg/kg) showed a greater neuroprotective effect. In conclusion, these findings suggest that CGA attenuates the ISO‐induced cognitive impairment via its anti‐inflammatory, anti‐oxidative, and anti‐apoptotic properties in aged mice.

## Graphical abstract

• Postoperative cognitive dysfunction (POCD), is characterized by impairment in cognitive functions in patients following anesthesia and surgery. • Chlorogenic acid (CGA) is a plant‐derived compound possessing numerous bioactive properties. • The aim of this study was to investigate the therapeutic potential of CGA in isoﬂurane (ISO)‐induced cognitive dysfunction of aged mice. • Our findings suggest that CGA attenuates the ISO‐induced cognitive impairment via its anti‐inflammatory, anti‐oxidative and anti‐apoptotic properties in aged mice.

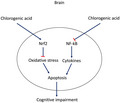

## 1 INTRODUCTION

Cognitive decline occurs in all persons during the aging process, and this eventually can result in mild cognitive impairment and dementia (Morley, 2018). Many elderly patients have the experience of postoperative cognitive dysfunction (POCD). POCD could prolong hospitalization, reduces quality of life, and also enhances the risk of death (Guo et al., 2019). It has been commonly accepted that POCD is linked to cognitive dysfunctions such as memory, attention, speech, and abstract thinking (Bekker & Weeks, 2003). Isoflurane (ISO), a general inhalation anesthetic used for induction and maintenance of general anesthesia, has been suggested to promote cognitive dysfunction in patients after surgery (Cui, Xu, & Qu, 2020; Jin et al., 2020). Although POCD is generally a well‐known disease, the precise molecular mechanism underlying its neurotoxicity remains largely unknown. Some factors including the age of patients, type of surgery, and the type of anesthesia have been reported to be involved in the onset of POCD (Chi, Li, Lin, Wang, & Zhou, 2017). In addition, neuroapoptosis, neuroinflammation, and oxidative stress have been demonstrated to be the important etiological factors in the development of POCD (Brambrink et al., 2010; Wu et al., 2012). Animal studies have suggested an increased inflammatory cytokines induction by anesthesia in the brain after surgery (Wang et al., 2018), and ISO‐induced cognitive dysfunction can be ameliorated by suppressing neuroinflammation (Zhang, Li, Li, Hou, & Hou, 2016; Zuo, 2013). In addition, ISO exposure can induce oxidative stress in the brain leading to initiation of neuron apoptosis and subsequently cognitive impairment (Shao, Wu, Bai, Fu, & Zou, 2019; Shen, Shang, Wu, & Ren, 2020). Therefore, any strategy to attenuate ISO‐induced neuroinflammation, neuroapoptosis, and oxidative stress might be helpful for preventing or treating POCD.

Chlorogenic acid (CGA), a type of phenolic acid, has been reported to have important biological actions, including anti‐apoptotic, anti‐inflammatory, anti‐oxidative, and anticancerous effects (Tajik, Tajik, Mack, & Enck, 2017). The neuroprotective property of CGA has also been demonstrated in several studies (Cropley et al., 2012; Kwon et al., 2010). Specifically, CGA has been reported to exert a neuroprotective role in Parkinson's disease (Heitman & Ingram, 2017; Singh et al., 2020). In addition, CGA acts as a potent neuroprotective agent by modulating the apoptotic‐related proteins in focal ischemia animal models (Shah, Kang, Park, Kim, & Koh, 2021). However, whether CGA can also improve the cognitive dysfunction induced by ISO remains unknown. Given the beneficial effects of CGA, here, we aimed to explore the ability of CGA to inhibit ISO‐induced cognitive impairment, neuroapoptosis, neuroinflammation, and oxidative stress in the hippocampus of aged mice.

## 2 MATERIALS AND METHODS

### 2.1 Animals

Aged C57BL/6 mice (20 months old) were purchased from the Animal Experiment Center of Sangon Biotechnology Co., Ltd. (Shanghai, China). All animals were kept under a 12 h dark/light cycle, 22 ± 1°C, and 55 ± 5% humidity with free access to water and standard rodent chow. All animal procedures were performed according to the Care and Use of Laboratory Animals by the National Institutes of Health.

After 1 week of adaptation, 80 mice were randomly divided into four groups: control group, isoflurane group (ISO), ISO + 30 mg/kg CGA group, and ISO + 60 mg/kg CGA (N = 20 in each group). Chlorogenic acid (Sigma‐Aldrich, purity 95%; St. Louis, MO, USA) was dissolved in sterile saline and administered orally by gavage once daily for 7 days, while the normal group was given sterile saline (Liu et al., 2021). On day 8, the mice were exposed to 1.5% ISO (Nanjing Jiancheng Bioengineering Institute), mixed with 100% O_2_ for 4 h, and the mice in the control group received 100% O_2_ of 1.5 L/min for 4 h in an identical chamber (X.‐m. Li et al., 2014). Immediately after the ISO exposure, arterial blood gases (ABG) and blood glucose levels were measured using a portable blood gas analyzer (OPTI Medical Systems, Georgia, USA) and glucose meter (Abbot Laboratories, USA), respectively. The mice (N = 10 for each group) were euthanized 24 h after ISO exposure for hippocampal harvesting and then biochemical analyses. One day after isoflurane exposure, 10 mice from each group were subjected to behavioral tests.

### 2.2 Behavioral study

#### 2.2.1 Open‐field test

We performed an open‐field test to study the emotional responses and exploration activity to a novel environment (Zhang, Li, Li, & An, 2016). The mice were placed in the center of the white plastic chambers. Each mouse was left to explore it for 5 min while the activity was recorded using a video tracking system (Shanghai Softmaze Information Technology Co., Ltd.). The parameters of total distance and the mice spent in the central area were recorded in this experiment.

#### 2.2.2 Morris water maze (MWM) experiment

To study the spatial learning and memory of mice, the MWM test was carried out as described previously (Yuan et al., 2019). In this test, a pool with a diameter of 120 cm and a depth of 50 cm was used. During the training, a cylinder platform was constant just below the surface of the circular pool. The swimming motions of the mice were recorded using a video tracking system. In each of the four quadrants of the swimming pool, all mice received four trials every day. Each mouse was allowed to find the platform within 60 s and the escape latency and mean speed were recorded. On the fifth day, the platform was removed and the same test was performed, and time spent in each quadrant and the number of platform crossing were measured.

### 2.3 Measurements of pro‐inflammatory cytokines

The protein levels of interleukin (IL)‐6, IL‐1β, and tumor necrosis α (TNF‐α) in the hippocampus were evaluated by using commercial enzyme‐linked immunosorbent assay kits (Novusbio, USA) according to the manufacturer's instruction.

### 2.4 Measurement of MDA, ROS, and SOD

The level of malondialdehyde (MDA), as a product of lipid peroxidation, and the activities of superoxide dismutase (SOD) and catalase (CAT) were measured in the hippocampus using commercial kits (Nanjing Jiancheng Co., Nanjing, China).

### 2.5 Real‐time PCR

Total RNA of the hippocampus was extracted using TRIzol (Invitrogen, California, USA). After synthesis of cDNA using a commercial kit, real‐time PCR was performed by TransStart Green q‐PCR SuperMix (TransGen Biotech, China). The following primers were used: NAD(P)H Quinone Dehydrogenase 1 (NQO‐1) forward, 5‐ GCCGAACACAAGAAGCTGGAAG‐3′; NQO‐1 reverse, 5′‐ GGCAAATCCTGCTACGAGCACT‐3′, heme oxygenase‐1 (HO‐1) forward, 5′‐ CCAGGCAGAGAATGCTGAGTTC‐3′, HO‐1 reverse: 5′‐ AAGACTGGGCTCTCCTTGTTGC‐3′, GAPDH forward, 50‐ CATCACTGCCACCCAGAAGACTG‐3′, and GAPDH reverse, 5′‐ ATGCCAGTGAGCTTCCCGTTCAG‐3′. The relative changes in mRNA expression were calculated by the 2^−ΔΔ^ method.

### 2.6 Western blot analysis

Cytoplasmic and nuclear proteins from the hippocampus tissues were isolated using a commercial protein extraction kit (Beyotime Institute of Biotechnology, Haimen, China). Protein concentrations were determined by the BCA Protein Assay Kit (Well‐bio, China). An equal amount of proteins was resolved by SDS‐PAGE and were subsequently transferred to a polyvinylidene fluoride (PVDF) membrane. After blocking with 10% skim milk in Tris‐buffered saline (TBS), the membranes were incubated with primer antibodies, nuclear factor erythroid factor 2‐related factor 2 (Nrf2) (Cell Signaling Technology, Boston, USA), p65 NF‐kB (Cell Signaling Technology, Boston, USA), IKBα (Cell Signaling Technology, Boston, USA), Lamin B1(Abcam, USA), Bcl‐2 Associated X (Bax) (Santa Cruz, USA), B‐cell lymphoma 2 (Bcl‐2) (Santa Cruz, USA), caspase 3 (Cell Signaling Technology, Boston, USA), and β‐actin (Sigma, USA). The membranes were then incubated with the secondary antibodies at room temperature for 2 h. Then, enhanced chemiluminescence (ECL) detection system (GE, Healthcare Life Sciences) was used to visualize immunoreactive proteins. The intensity of each band was quantified with the densitometric analyses (Bio‐Rad Laboratories).

### 2.7 Statistical analysis

SPSS version 25 (SPSS, Chicago IL) was used for statistical analysis. The one‐way ANOVA followed by Tukey post‐hoc test was applied to compare the differences between the groups. The data are presented as mean ± SD. Graphs were drawn using GraphPad Prism version 8. Values of *p* < .05 were also considered significant.

## 3 RESULTS

### 3.1 Physiological parameters after isoflurane exposure

We did not find significant differences in the levels of ABG parameters and blood glucose concentrations among treatment groups immediately after the 4 h exposure to 1.5% ISO (Table 1). These findings suggest that ISO‐induced neurodegeneration in the hippocampus was not due to the physiological side effects.

**TABLE 1:** Physiological parameters immediately after the 4 h exposure to 1.5% isoflurane

Group	pH	PaCO2 (mmHg)	PaO2 (mmHg)	SaO2 (%)	Glucose (mmol/L)
Control	7.39 ± 0.04	38.4 ± 1.8	164 ± 6.9	98 ± 2.9	5.0 ± 0.9
ISO	7.42 ± 0.07	39.3 ± 2.3	159 ± 11.2	97 ± 2.1	5.1 ± 0.9
ISO + 30 mg/kg CGA	7.36 ± 0.05	37.9 ± 2.9	162 ± 9.3	97 ± 2.0	5.2 ± 0.8
ISO + 60 mg/kg CGA	7.38 ± 0.04	37.5 ± 4.2	163 ± 5.6	97 ± 1.9	5.1 ± 0.6
*p*‐Value	NS	NS	NS	NS	NS

*Note:* Values are given as mean ± SD.

Abbreviations: PaCO2, arterial carbon dioxide tension; PaO2, arterial oxygen tension; SaO2, arterial oxygen saturation; NS, non‐significant (*n* = 10 for each group).

### 3.2 Chlorogenic acid attenuated cognitive impairment induced by isoflurane

**FIGURE 1:**
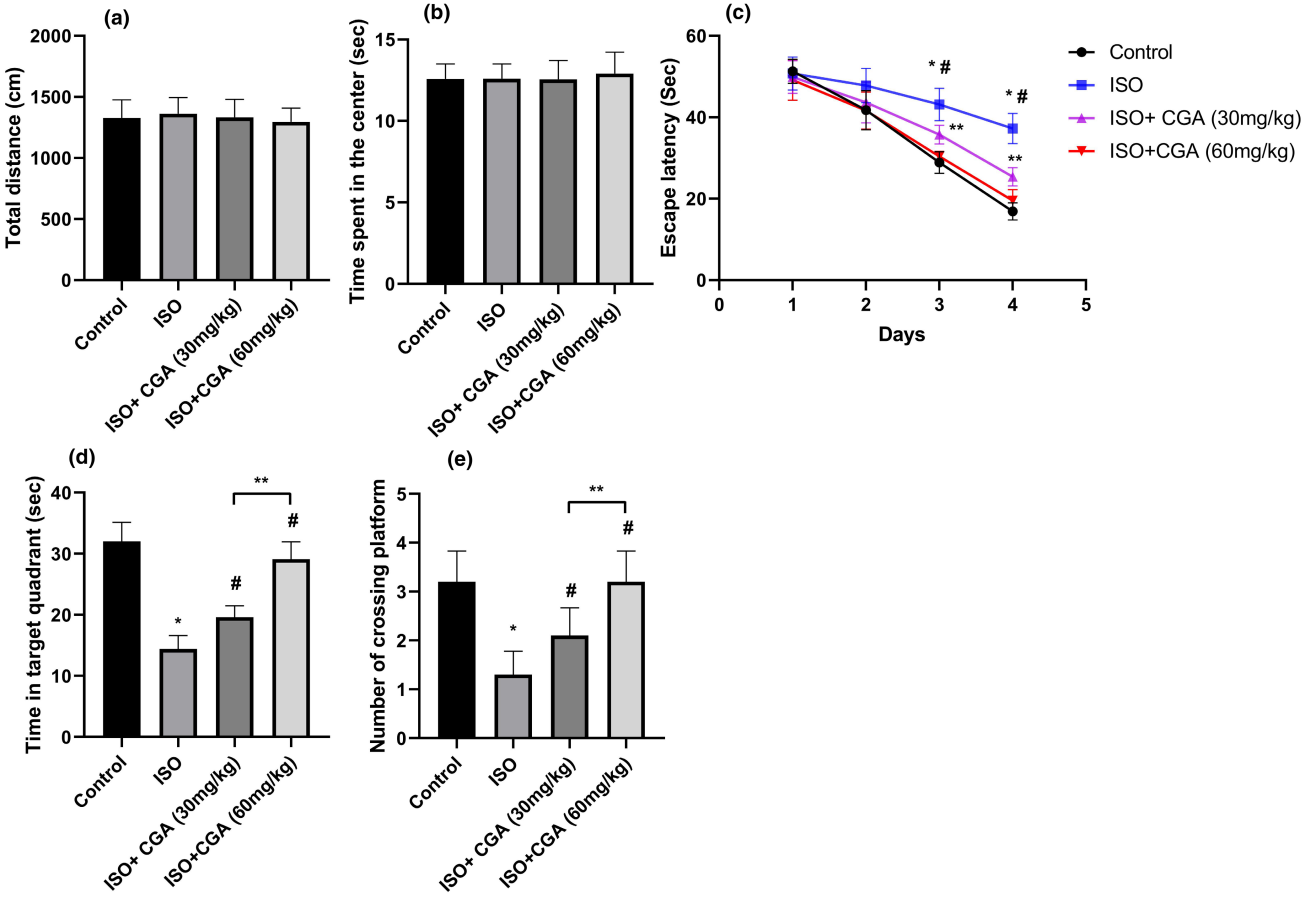
The effects of CGA pretreatment on isoflurane‐induced cognitive impairment in aged mice. (a, b) The total distance and the time in the center of the open field. The escape latency (c), time spent in the target quadrant (d), and the number of platform crossings (e) of mice in the MWM test. The values are represented as mean ± SD, (*n* = 10 per group). *p < .05 control group vs. ISO group, #*p* < .05 ISO vs. ISO + 30 mg/kg CGA and ISO + 60 mg/kg CGA groups, and **p < .05 ISO + 30 mg/kg CGA group vs. ISO + 60 mg/kg CGA group

The data of the total travel distance and the time spent in the center of the arena in the open‐field test revealed no significant difference between the groups (Figure 1a,b), suggesting that the ISO exposure had no significant impact on the locomotor and exploratory activities. Furthermore, CGA treatment with the two doses (30 and 60 mg/kg) did not alter the general behavioral performance.

To investigate the influence of CGA pretreatment on ISO‐induced deficits in memory and spatial learning activities, the MWM test was performed. Isoflurane exposure led to an enhancement in the escape latency (Figure 1a) and a decrease in the target quadrant time (Figure 1b) and cross‐platform times (Figure 1c) in comparison with those of the control group. However, both the 30 and 60 mg/kg CGA treatments significantly attenuated the changes in the escape latency during the training period, the time in the target quadrant, and cross‐platform time. Importantly, it appears that CGA treatment suppresses ISO‐induced cognitive impairment in a dose‐dependent manner. A higher dose (60 mg/kg) of CGA fully restored the latency to find the platform, the time in the target quadrant, and cross‐platform times similar to control mice, whereas lower CGA dose (30 mg/kg) partially restored these parameters. There were no differences between control, ISO + CGA (30 mg/kg), and ISO + CGA (60 mg/kg) groups in the MWM test. Taken together, these findings suggest that the memory impairment after ISO exposure can be reversed by CGA treatment in mice.

### 3.3 Chlorogenic acid attenuated ISO‐induced neuroinflammation

**FIGURE 2:**
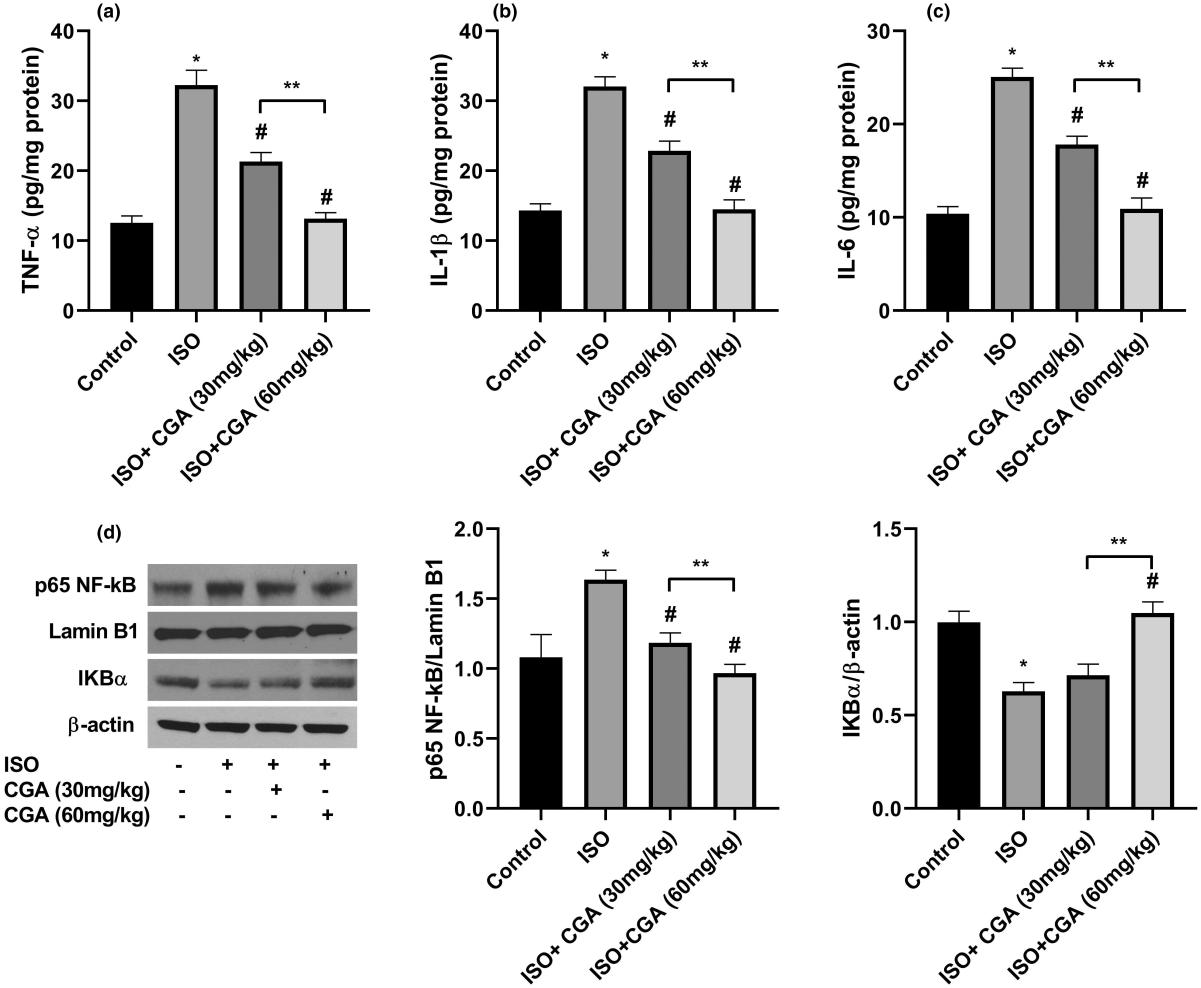
The effects of CGA pretreatment on neuroinflammation of ISO‐exposed aged mice. Hippocampi were homogenized and the levels of TNF‐α (a), IL‐1β (b), and IL‐6 (c) were measured. (d) The nuclear NF‐κB and the cytoplasmic IκBα were determined by western blot analysis. Data are represented as mean ± SD. *p < .05 control group vs. ISO group, #*p* < .05 ISO vs. ISO + 30 mg/kg CGA and ISO + 60 mg/kg CGA groups, and **p < .05 ISO + 30 mg/kg CGA group vs. ISO + 60 mg/kg CGA group

Isoflurane exposure has been reported to induce neuroinflammation (Shi et al., 2017). To study the potential anti‐inflammatory role of CGA in ISO‐exposed mice, we detected the levels of hippocampal pro‐ inflammatory cytokines. We observed significantly greater levels of IL‐6, IL‐1β, and TNF‐α upon ISO exposure. Chlorogenic acid treatment was able to attenuate ISO‐induced pro‐inflammatory cytokines production, whereas the higher concentration of CGA (60 mg/kg) completely restored these levels to that of control mice (Figure 2a–c). We also evaluated the levels of p65 NF‐kB and IKBα, two major components of the NF‐κB inflammatory pathway. The ISO‐exposed group had an increased level of p65 NF‐κB in the nucleus and downregulated level of IκBα in the cytoplasm compared with the control group. CGA treatments significantly reversed these changes in the ISO group (Figure 2d), whereas a higher inhibitory effect was observed in the CGA (60 mg/kg) group.

### 3.4 Chlorogenic acid attenuated ISO‐induced oxidative stress

**FIGURE 3:**
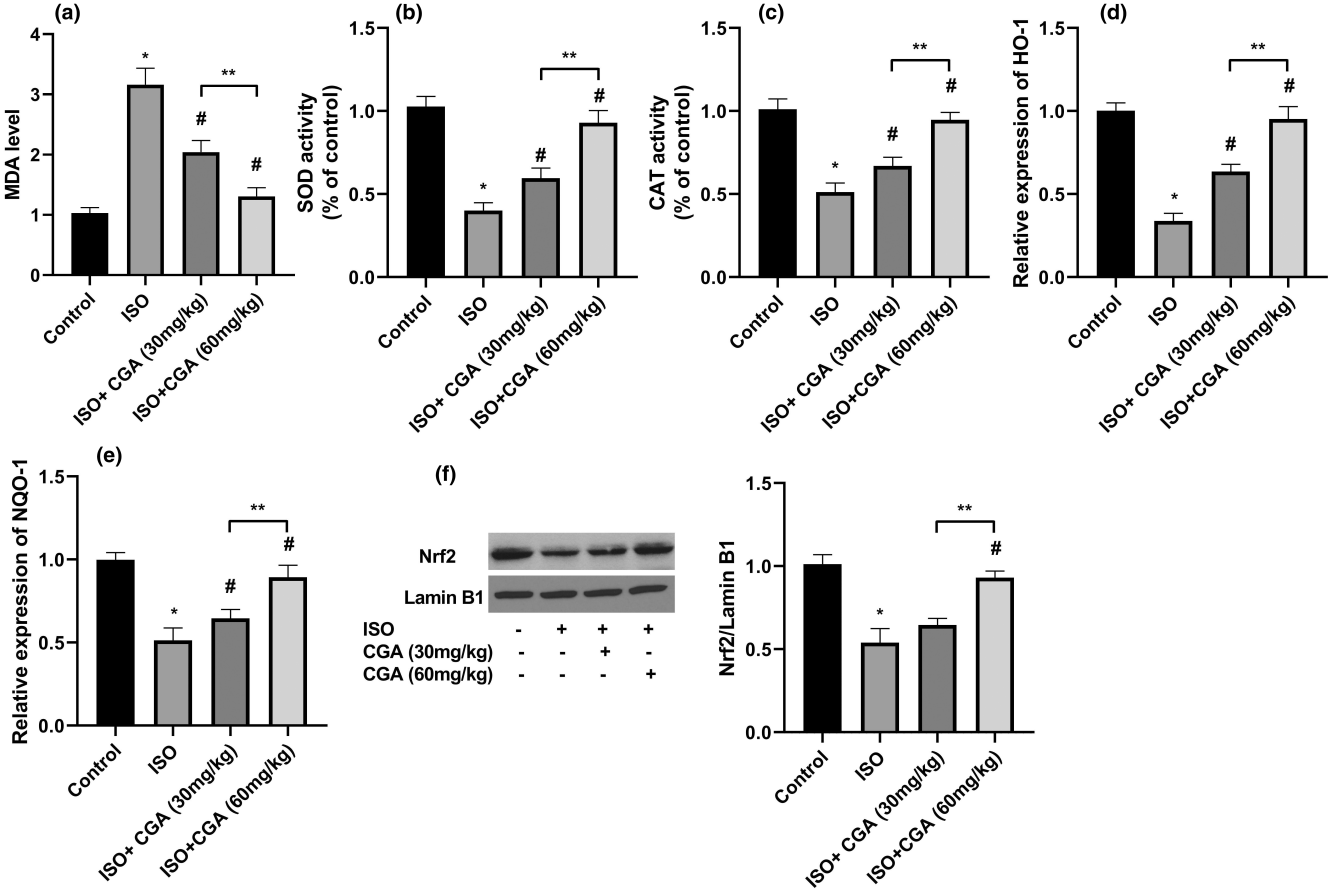
The effects of CGA pretreatment on oxidative stress in the hippocampus of ISO‐exposed aged mice. The MDA (a) SOD (b), and CAT (c) levels were measured in the hippocampus of different groups. The expression of HO‐1 (d) and NQO‐1 (e) were measured by real‐time PCR. The nuclear (f) Nrf2 was determined by western blot analysis. Data are represented as mean ± SD. *p < .05 control group vs. ISO group, #*p* < .05 ISO vs. ISO + 30 mg/kg CGA and ISO + 60 mg/kg CGA groups, and **p < .05 ISO + 30 mg/kg CGA group vs. ISO + 60 mg/kg CGA group

To evaluate the effect of ISO exposure on oxidative stress in the hippocampus, we examined the levels of several markers of oxidative stress. As shown in Figure 3a–c, there were significant increase in the MDA level and an efficient decrease in the activities of SOD and CAT following ISO exposure in mice compared with the control. The MDA level was lower and SOD and CAT activities were higher in both CGA groups than those in the ISO group. Importantly, the levels of MDA and the activities SOD and CAT in ISO + CGA (60 mg/kg) group were similar to the control group.

We next focused on the Nrf2/ARE pathway, an important signaling pathway that confers protection to various oxidative stress‐related diseases (Ma, 2013). The nucleus Nrf2 was downregulated in the ISO group in comparison with control. Importantly, CGA treatments up‐regulated the expression of Nrf2 in the nucleus, whereas ISO + CGA (60 mg/kg) treatment completely restored nuclear Nrf2 level to that of control mice (Figure 3e). Moreover, we evaluated the expression of Nrf2‐target genes, HO‐1, and NQO‐1 by real‐time q‐PCR. The results revealed the suppression of both genes expression following ISO exposure (Figure 3c,d). We observed significant upregulation of HO‐1 and NQO‐1 genes in CGA groups in comparison with the ISO group.

### 3.5 Chlorogenic acid attenuated ISO‐induced neuroapoptosis

**FIGURE 4:**
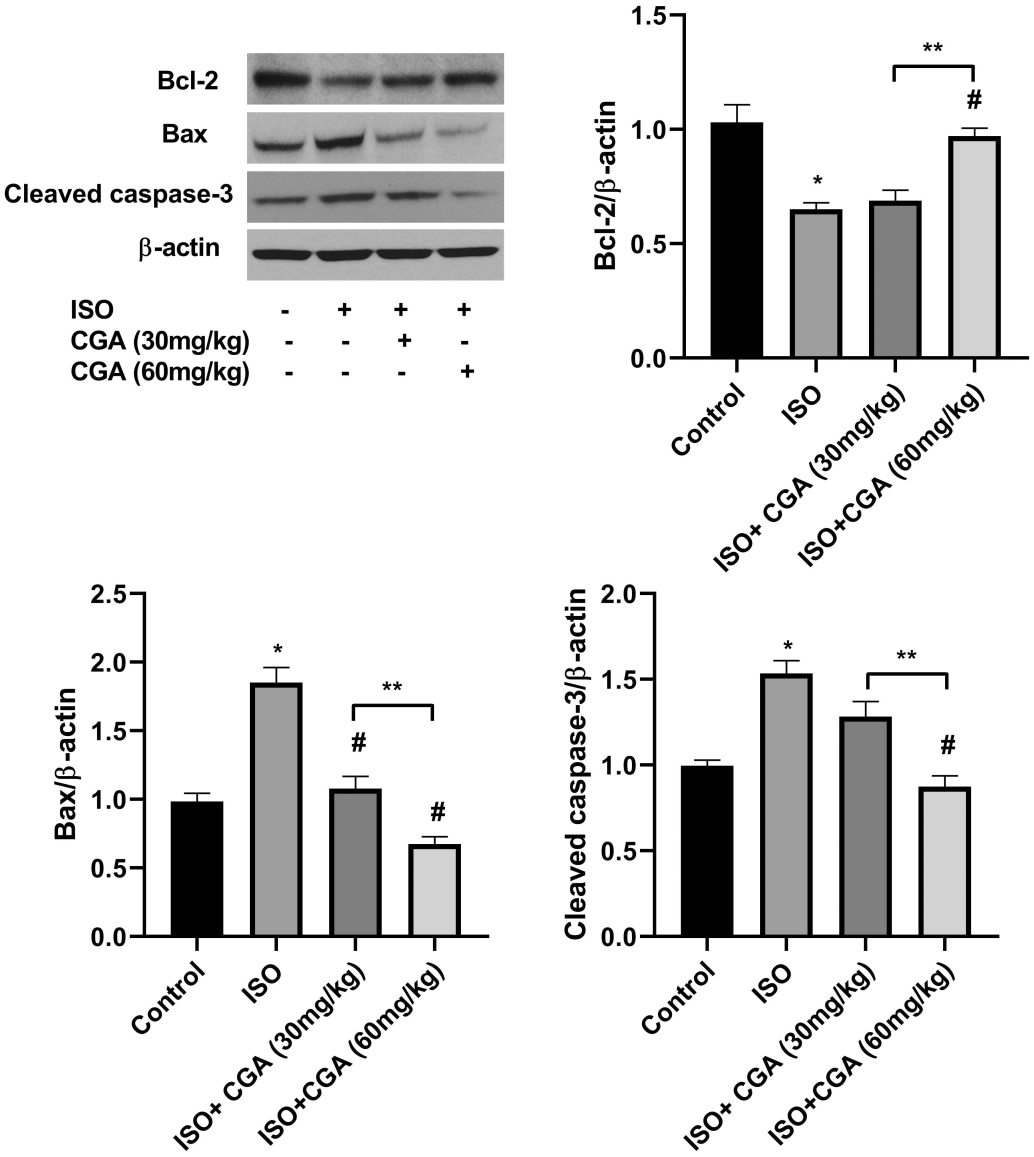
The effects of CGA pretreatment on apoptosis in the hippocampus of ISO‐exposed aged mice. The levels of Bcl‐2, Bax, and cleaved caspase‐3 were determined by western blot analysis. Data are represented as mean ± SD. *p < .05 control group vs. ISO group, #*p* < .05 ISO vs. ISO + 30 mg/kg CGA and ISO + 60 mg/kg CGA groups, and **p < .05 ISO + 30 mg/kg CGA group vs. ISO + 60 mg/kg CGA group

The results of immunoblotting showed that protein levels of the cleaved caspase‐3 and Bax enhanced and Bcl‐2 reduced following ISO exposure in comparison with the control group (Figure 4a–c). Chlorogenic acid (60 mg/kg) pretreatment significantly mitigated the ISO‐induced enhancement of cleaved caspase‐3 and Bax, and reduction of Bcl‐2 after the ISO anesthesia, whereas CGA 30 mg/kg partially affects these parameters.

## 4 DISCUSSION

Isoflurane, a widely used volatile anesthetic, was suggested to promote neurodegeneration and cognitive impairments in aged rodents (Cao, Li, Lin, & Zuo, 2012; Li et al., 2014). Similar to previous studies, we observed cognitive impairments following exposure of old mice to 1.5% ISO for 4 h. To exclude the effects of hypoglycemia and hypoxia on cognitive functions during ISO anesthesia, ABG analysis and blood glucose levels were evaluated at the end of the ISO exposure. The data demonstrated no significant alteration in any of these parameters, indicating no physiological side‐effects during ISO‐induced cognitive impairments. Neuroinflammation, neuroapoptosis, and oxidative stress have been found to play important roles in cognitive dysfunction after surgery or anesthesia (B. Li et al., 2018; Terrando et al., 2011; Zhang et al., 2012). Consistently with these findings, we demonstrated that the exposure to ISO induces inflammation, apoptosis, and oxidative stress in the hippocampus of the aged mice.

The main focus of the current study was to detect the neuroprotective effects of CGA in ISO‐induced cognitive deficits in vivo. To evaluate the effects of CGA on cognitive function after ISO exposure, the open‐field test and Morris water maze test were conducted. Our data revealed that CGA attenuates learning and memory decline induced by 1.5% ISO in aged mice, as demonstrated by the shorter escape latency, the longer times of original platform crossing, and the more time spent in the target quadrant in the Morris water maze test. We also observed that CGA pretreatment in ISO‐exposed mice restores cognitive function in a dose‐dependent manner and 60 mg/kg CGA completely restored cognitive function in aged mice. In agreement with above findings, treatment with CGA significantly attenuated the scopolamine‐induced memory impairments in rats (Fukutomi et al., 2021; Jang et al., 2013; Kwon et al., 2010). In addition, Ishida et al. reported that chronic administration of CGA attenuated cognitive impairments and prevented Aβ deposition and neuronal loss in APP/PS2 transgenic mouse model of Alzheimer's disease (Ishida et al., 2020).

The evidence has suggested that neuroinflammation has a critical role in the development of cognitive dysfunction (Kong, Ma, Zhang, & Zhou, 2015; Terrando et al., 2011). To investigate the molecular mechanisms underlying the neuroprotective effects of CGA, we have focused on the neuroinflammation of ISO‐exposed mice. We found that CGA pretreatment was able to ameliorate ISO‐induced upregulation of pro‐inflammatory cytokines IL‐6, IL‐1β, and TNF‐α in a dose‐dependent manner. We also evaluated the levels of nuclear p65 NF‐kB and cytoplasmic IκBα, two major components of the NF‐κB inflammatory pathway. We observed that the activation of the NF‐kB pathway was suppressed following pretreatment of CGA in aged mice. In support of these findings, Singh et al. have reported that CGA can decrease drug‐induced neuroinflammation in substantia nigra, and this effect was mediated through regulating the NF‐κB expression and production of pro‐inflammatory cytokines such as TNF‐α and IL‐1β along with upregulation of anti‐inflammatory cytokine IL‐10 (Fukutomi et al., 2021; Singh et al., 2020).

Oxidative stress was reported to be involved in the progress of neurodegeneration after surgery undergoing volatile anesthetics (Yalcin et al., 2013). In oxidative stress state, the imbalance between generation of reactive oxygen species (ROS) and antioxidant defenses system activity leads to excessive accumulation of ROS and subsequently the death of neurons and synapses, and then development of cognitive deterioration (Kwon et al., 2010). MDA, the final product of lipid peroxidation, is widely used as a marker of oxidative damage. Superoxide dismutase and CAT are enzymes that protect against oxidative stress by eliminating oxygen free radicals (Huang, Zou, & Corniola, 2012). The nuclear factor erythroid 2–related factor 2 (Nrf2) is a critical regulator of cellular resistance to oxidants through regulating a group of antioxidant response element–dependent genes such as SOD, CAT, HO‐1, and NQO‐1 (Ma, 2013). In this regard, the evidence has suggested that activating the Nrf2/ARE signaling pathway may attenuate neurotoxicity (Piras et al., 2017). To explore whether the neuroprotective effects of CGA are mediated through inhibiting the oxidative stress in aged mice, several markers were evaluated. CGA pretreatment, particularly at a higher dose (60 mg/kg CGA), significantly reversed the downregulation of SOD and CAT activities and reduced MDA level in the hippocampus of the ISO‐exposed mice. Furthermore, our data demonstrated that CGA promoted the nuclear translocation of Nrf2 and subsequently elevated the expression of its downstream target genes HO‐1 and NQO1 in the hippocampus of mice exposed to ISO. These findings imply that CGA might protect cognitive impairments induced by ISO via activating the Nrf2/ARE signaling pathway. In agreement with our data, a recent study showed that trilobatin, a glycosylated dihydrochalcone derived from the leaves of *L. polystachyus* Rehd, prevented ISO‐induced neurotoxicity in HT22 cells via activating the Nrf2/ARE pathway (Shen et al., 2020). In another study, Shi et al. demonstrated that CGA restrains the apoptosis of Aβ 25–35‐induced hippocampal neurons via reducing the activities of SOD, and glutathione peroxidase (GSH)‐Px and MDA level (Shi et al., 2020).

Previous studies have reported neuroapoptosis in the hippocampi following exposure to ISO. Hippocampal neuronal apoptosis was associated with Bcl‐2 downregulation, Bax upregulation, and increased cleaved caspase‐3 levels (Hua et al., 2016). In the present study, we observed that CGA pretreatment dose‐dependently reversed the effect of ISO exposure on Bcl‐2 downregulation, Bax upregulation, and increased cleaved caspase‐3, thereby inhibiting apoptosis in the hippocampus region. Singh et al. found similar results that CGA significantly inhibited the activation of proapoptotic proteins including Bcl‐2 and caspase‐3 and elevation of anti‐apoptotic protein such as Bcl‐2 in the brain of 1‐methyl‐4‐phenyl‐1,2,3,6‐tetrahydopyridine (MPTP)‐induced mice model of Parkinson's disease (Singh et al., 2020).

In summary, the data of the present study suggest that CGA pretreatment in ISO‐exposed mice restores cognitive function in a dose‐dependent manner. CGA suppresses neuroinflammation through inhibiting pro‐inflammatory cytokines production and NF‐κB signaling pathway. In addition, CGA could inhibit oxidative stress via activating the Nrf2 signaling pathway and eventually inhibiting apoptosis in the hippocampus of aged mice exposed to ISO. Strikingly, 60 mg/kg CGA completely restored neuroinflammation, neuroapoptosis, and oxidative stress in mice exposed to ISO. These findings provide the evidence that CGA use might be a preventive strategy for ISO‐induced cognitive dysfunction in the clinic.

## CONFLICT OF INTEREST

The authors have nothing to declare.
